# Revealing the influence of electron beam melted Ti-6Al-4V scaffolds on osteogenesis of human bone marrow-derived mesenchymal stromal cells

**DOI:** 10.1007/s10856-021-06572-0

**Published:** 2021-08-18

**Authors:** Kristin S. Ødegaard, Lingzi Ouyang, Qianli Ma, Glenn Buene, Di Wan, Christer W. Elverum, Jan Torgersen, Therese Standal, Marita Westhrin

**Affiliations:** 1 Department of Mechanical and Industrial Engineering, Norwegian University of Science and Technology, Trondheim, Norway; 2 Department of Clinical and Molecular Medicine, Norwegian University of Science and Technology, Trondheim, Norway

## Abstract

Porous Titanium-6Aluminum-4Vanadium scaffolds made by electron beam-based additive manufacturing (AM) have emerged as state-of-the-art implant devices. However, there is still limited knowledge on how they influence the osteogenic differentiation of bone marrow-derived mesenchymal stromal cells (BMSCs). In this study, BMSCs are cultured on such porous scaffolds to determine how the scaffolds influence the osteogenic differentiation of the cells. The scaffolds are biocompatible, as revealed by the increasing cell viability. Cells are evenly distributed on the scaffolds after 3 days of culturing followed by an increase in bone matrix development after 21 days of culturing. qPCR analysis provides insight into the cells’ osteogenic differentiation, where *RUNX2* expression indicate the onset of differentiation towards osteoblasts. The *COL1A1* expression suggests that the differentiated osteoblasts can produce the osteoid. Alkaline phosphatase staining indicates an onset of mineralization at day 7 in OM. The even deposits of calcium at day 21 further supports a successful bone mineralization. This work shines light on the interplay between AM Ti64 scaffolds and bone growth, which may ultimately lead to a new way of creating long lasting bone implants with fast recovery times.

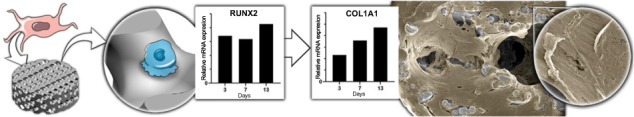

## Introduction

Titanium-6 Aluminum-4 Vanadium (Ti64) is a common biomaterial due to its high strength-to-weight ratio, biocompatibility, good corrosion and fatigue resistance [1, 2]. However, the stiffness is too high compared to human bone leading to stress shielding, the major cause of implant loosening [3–5]. Through tailored porosity, the elastic modulus can be modified, and bone ingrowth into the implant can be facilitated, further increasing the bone-to-implant interlocking. Such tailored geometries can be defined by computer aided design (CAD) and manufactured by powder bed-based additive manufacturing (AM), either selective laser melting (SLM) or electron beam melting (EBM) [6]. Ti64 samples manufactured by SLM are characterized to have higher yield- and ultimate tensile strength, but lower ductility compared to samples manufactured with EBM [7]. Due to the FDA-approval of EBM, the EBM process also sets the current benchmark in this field [8, 9].

Bone remodeling around the implant is a complex process, and the success of implantation cannot be described by a good interlock alone. The as-built surface of such implanted porous scaffolds is generally rough, characterized by dome-shaped, partly sintered particles with surface roughness ranging between *R*_*a* _= 1 µm and 40 µm [10, 11]. The topological effect of the scaffold material on the cells can be characterized by (1) interactions with the rough surfaces and (2) interactions with the macroscopically designed porosity.

The influences of surface texture on cell fate (1), have been thoroughly investigated. Researchers have shown that rough titanium surfaces with irregular morphologies result in higher levels of cellular attachment and enhanced osteoblast differentiation [12–15]. Considering (2), studies on 3D porous designs are generally limited to investigating bone ingrowth in vivo. We hence focus on how the combination of designed porosity and surface texture affects osteogenesis in vitro.

Although research has been conducted to investigate bone ingrowth on AM Ti64 scaffolds, most have employed osteoblast-like cell lines, typically isolated from bone tumors. These cell lines are good candidates for examining cell migration and osteoblast differentiation; however, they have inherent instability and possibly aberrant behavior [16]. Such cell lines can also contain a heterogeneous mixture of osteoblastic cells at different stages of differentiation [17]. To better represent the in vivo situation, mesenchymal stromal cells (MSCs) are a superior choice [18]. These cells have the ability to differentiate into osteoblasts from the quiescent state in vivo [19, 20]. By further inducing the cells to enter the quiescent state in vitro, the stemness phenotype, including osteoblast differentiation, can be enhanced [21].

In this study, we characterize the activity, differentiation, matrix formation and mineralization of bone marrow-derived MSCs (BMSCs) cultured on EBM manufactured scaffolds of 800 µm void spacing and lattice thickness, constituting a typical implant design reported to favor bone ingrowth [22, 23]. We provide insight into cell viability revealed by AlamarBlue assays, cell adhesion and matrix formation through microscopic imaging, osteogenic differentiation by mRNA expression of specific genes (*RUNX2*, *COL1A1*, *BGLAP* and *SOST*) and bone mineralization by alkaline phosphatase activity and calcium staining. Beyond cell activity and bone ingrowth, this study gives further insight into the potential of AM porous Ti64 scaffolds for use in bone implants.

## Materials and methods

### Scaffold fabrication and preparation

**Fig. 1:**
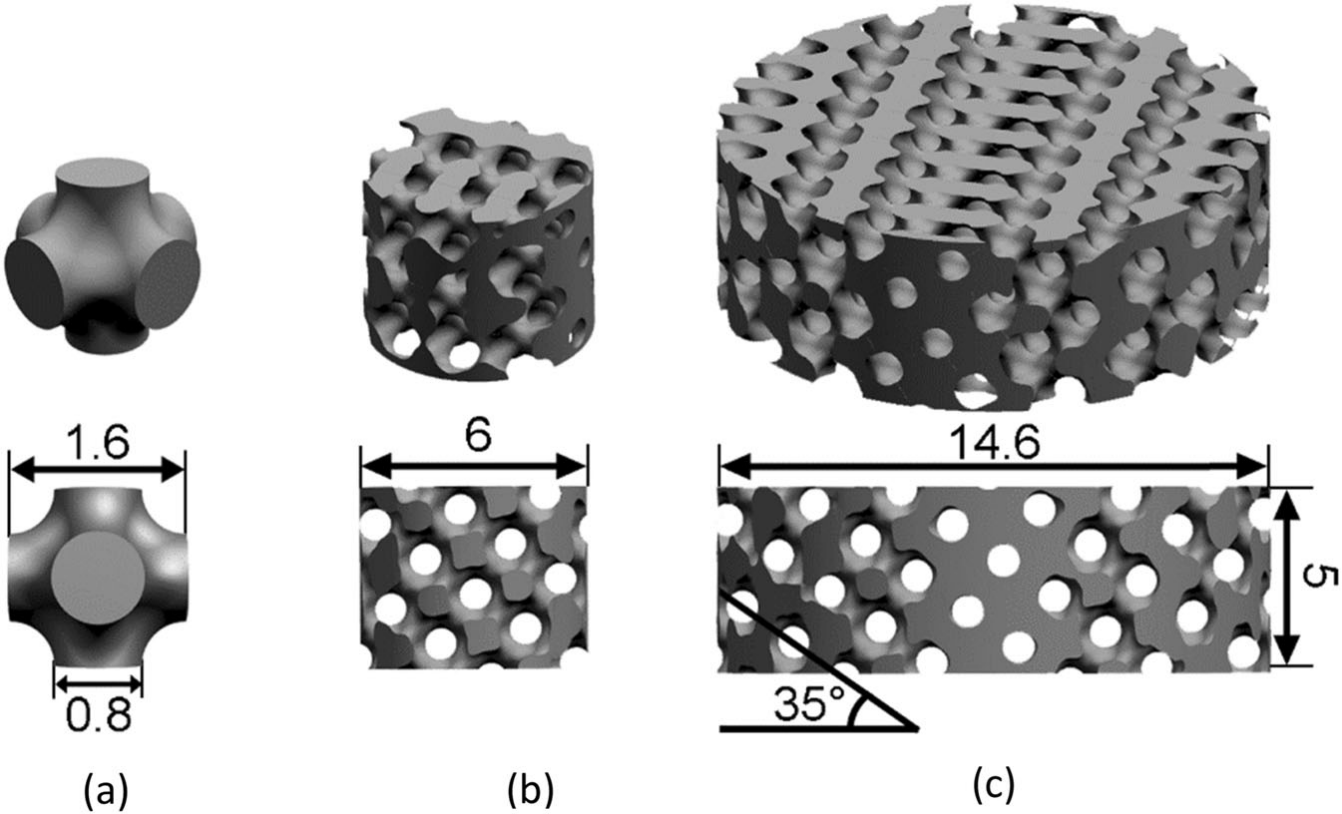
CAD designs used for scaffold manufacturing. Measurements are in millimeters

Lattice-type scaffolds were designed based on the Schwarz P triply periodic minimal surface-based unit cell. Lattice- and pore diameter were set to 800 µm giving a designed porosity of 50%. Siemens NX (Siemens PLM Software, Germany) was used to design the CAD-model. Figure 1a shows the CAD-model of the lattice unit cell that was repeated throughout the scaffold. The unit cells were directed in a 35° angle. Scaffolds were designed having a height of 5 mm and a diameter of 6 mm (small scaffold, Fig. 1b) and 14.6 mm (large scaffold, Fig. 1c). Each design fits into a 96- (small) and 24- (large) well plate, respectively. The scaffolds were manufactured by FIT AG (Germany) using Electron Beam Melting (EBM, Arcam Q10, GE Additive, Sweden) utilizing the powdered alloy Ti64 grade 5 as raw material. The process parameters used for the manufacturing are described in a previously published paper [24].

To evaluate the build accuracy of the manufactured parts, a small scaffold was imaged using a micro-computerized tomography (CT) scan (SkyScan 1176, Bruker microCT, Belgium). Cu + Al filter was used with the following acquisition settings: voltage 80 kV, current 312 µA, rotation step 0.30°, and exposure 1360 ms. The scans were reconstructed using NRECON (version 1.7.1.0, Bruker, Belgium), and analyzed using CTAn (version 1.17.8.0, Bruker, Belgium).

The scaffolds were sterilized using an ultrasonic (US) bath in several steps. First, the scaffolds were soaked in beakers containing 96% ethanol, and placed in the US bath (50 °C, 10 min, 37 fHz with sweep function, Elmasonic P, Elma Schmidbauer GmbH, Germany). Next, the scaffolds were cleaned with deionized water in the US bath (50 °C, 10 min, 37 fHz with sweep function). Finally, the scaffolds were heat treated (120 °C, 20 min).

### Cell culture

BMSCs from two healthy donors were used in the experiments. Donor 1 (D_1_), adult (Lonza Walkersville Inc, US), and Donor 2 (D_2_), child (BMSCs harvested for routine diagnostic purposes). The cells were maintained in growth medium (GM), consisting of Minimum Essential Media (Product no. 41061-029, Thermo Fisher Scientific, Norway) supplemented with gensumycine (5000 IU mL^−1^), glutamine (2 mm) and fetal bovine serum (10%). The cells were seeded at a density of 10^4^ cells cm^−2^ surface area. The scaffolds were placed in ultra-low attachment well plates (Product no. CLS3473 and CLS3474, Merck, Norway). The total amount of cells was calculated considering the CAD surface area of the scaffold design. To ensure an even distribution of cells on the scaffolds, 50% of the cells were seeded onto the scaffolds and incubated (1 h, 37 °C, 5% CO_2_), thereafter the scaffolds were flipped, and the rest of the cells were seeded. When the cells were seeded on the scaffolds, D_1_ was at passage 5 and D_2_ was at passage 7. The cells were initially seeded onto the scaffolds with regular GM and incubated (20 h, 37 °C, 5% CO_2_). The cells were then induced to quiescence by replacing the medium with serum-free medium, which consisted of the basal media, 5000 IU mL^−1^ gensumycine, and 2 mm glutamine, and incubated (4 h, 37 °C, 5% CO_2_) before stimulation with osteogenic media (OM) or GM. OM consisted of GM with L-ascorbic acid (50 µg mL^−1^, product no. A4403, Merck, Norway), dexamethasone (10 nm, product no. D2915, Merck, Norway) and *β*-glycerophosphate (10 mm, product no. G9422, Merck, Norway). The medium was changed every 2–3 days.

### Cell viability

Cell viability was measured using AlamarBlue™ Cell Viability Reagent (Product number DAL1100, Thermo Fisher Scientific, Norway) according to the manufacturer’s instructions. In brief, for each time point, AlamarBlue reagent was added to the culturing medium (1:10), and incubated (2 h, 37 °C). Next, the supernatant was harvested, and fresh medium was added. The fluorescence was measured in black 96-well plates on a multilabel fluorescence reader (Victor3, PerkinElmer, US). Samples were run in triplicates.

### Imaging

Cell adhesion and bone formation were examined for both donors using scanning electron microscope (SEM) and environmental SEM (ESEM). Imaging was performed separately after 3 and 21 days of culturing for both groups (OM and GM), respectively. Before imaging, the medium was removed, followed by 2–4 washing cycles with Dulbecco’s phosphate buffered saline (PBS, product no. D8537, Sigma-Aldrich, Germany). Cells were subsequently fixed with paraformaldehyde (4%, PFA, product no. 43368, Alfa Aesar, Germany) in PBS for 15 min at room temperature (RT). The scaffolds were then rinsed with PBS four times (RT). After fixation, cells and matrix were dehydrated in increasing concentrations of ethanol in distilled H_2_O (30, 50, 60 and 90%, 2 × 5 min, product no. 20824.296, VWR Chemicals, Norway). Finally, the scaffolds were put in ethanol with 96% concentration for 5 min 3 times. Scaffolds at day 3 were directly imaged after dehydration with ESEM (Quanta FEG 650 ESEM, ThermoFisher Scientific, Norway). Images were taken using a large field, low vacuum detector at a working distance of about 7 mm, voltage of 5 kV with an aperture of 30 µm and spot size of 3.0. Scaffolds at day 21 were sputter coated after dehydration prior to SEM imaging (4 nm, Pt/Pd target, 80:20 ratio, Cressington 208 HR, Cressington Scientific Instruments, England). The samples were imaged using SEM (Apreo SEM, ThermoFisher Scientific, Norway), with 0.8 nA current and 10 kV voltage. The Everhart-Thornley detector was employed in the secondary electron mode.

### Quantitative real-time PCR

Cells from D_1_ were seeded on eight large scaffolds and used for qPCR analysis after the culturing period. Four scaffolds were cultured in OM and four were cultured in GM. Cells were harvested at four different time points (Day 3, 7, 13, and 21) in both culture conditions (OM and GM). The cells were lysed directly on the scaffold using a lysis buffer (500 µL, Catalog no. 79216, QIAGEN, Sweden) and RNA was subsequently isolated using an RNA isolation kit (Catalog no. 74106, QIAGEN, Sweden) with DNase digest (Catalog no. 79256, QIAGEN, Sweden). RNA was eluted in RNase-free water and the RNA concentration was measured using a spectrophotometer (NanoDrop 1000, Thermo Fisher Scientific, US). Complementary DNA (cDNA) was synthesized by reverse transcription using a High-Capacity RNA-to-cDNA Kit (Catalog no. 4387406, Thermo Fisher Scientific, Norway). qPCR analysis was performed using the real-time PCR system (StepOnePlus Real-Time PCR System, Thermo Fisher Scientific, Norway). 3 ng of cDNA was used for qPCR, and each sample was measured in triplicates. The following probes were used for qPCR: Runt-related transcription factor 2 (*RUNX2*, Hs00231692_m1), collagen type I alpha 1 (*COL1A1*, Hs00164004_m1), osteocalcin (*BGLAP*, Hs01587814_g1), sclerostin (*SOST*, Hs00228830_m1), and GAPDH (Hs99999905_m1) as endogenous control. All probes were purchased from TaqMan, Applied biosystems, US. A cut-off value of 35 for the cycle threshold (Ct) was chosen.

### Calcium deposition

The calcium deposition of osteoblasts was evaluated using Alizarin Red staining (ARS, Catalog no. A5533, Merck, Norway). BMSCs from D_1_ were cultured on two small scaffolds in OM and GM, respectively, as indicated. For reference, cells were also cultured on a tissue culture polystyrene well plate (TCPS) in both OM and GM. At day 28, the cells were fixed as previously described. Prior to ARS staining, the scaffolds were washed with PBS and distilled *H*_2_*O,* ﻿respectively. The scaffolds were incubated with ARS (40 mm, 1 h, RT). To control for unspecific staining, a sterile cell-free scaffold was used as control. Images were taken with an automated imaging system (EVOS FL Auto 2, Thermo Fisher Scientific, US). After imaging, the scaffolds were destained using 10% wt vol^−1^ cetylpyridinium chloride in sodium phosphate buffer (10 mm, pH = 7, 1 h, RT). The supernatant was transferred to a 96-well plate and scanned in Microplate Absorbance Reader in triplicates (570 nm, 30 µL, iMark, Bio-rad Laboratories, US). The relative absorbance was calculated with respect to the control sample.

### Alkaline phosphatase activity

The alkaline phosphatase (ALP) activity was detected using the ELF Endogenous Phosphatase Kit (Product number E6601, Thermo Fisher Scientific, Norway). BMSCs from donor 1 were seeded on small scaffolds and cultured in OM and GM. Cells were fixed at day 7 as described earlier. Prior to staining, the cells were permeabilized with Tween-20 (0.2%, product no. P1370, Sigma-Aldrich, Germany) in PBS (10 min, RT). Subsequently, the scaffolds were rinsed in PBS (10 min, RT). The PBS was removed, and 50 µL per sample of the ELF97 mix (1:20 ELF97 to detection buffer) was added to each sample and incubated (15 min, RT). The samples were rinsed three times with PBS for a total time of 10–15 min with gentle agitation. PBS was then removed, and nuclei were stained with Hoechst 33342 (Product no. 62249, Thermo Fisher Scientific, Norway) in 1X dye solution and incubated (10 min, RT). The scaffolds were then taken out of the well plate, flipped upside down, and imaged using a high precision glass dish (Part no. P35G-0.170-14-C, MatTek, US). The fluorescence signal was then visualized using a confocal light microscope (Leica SP8, Leica Microsystems, Germany).

The dyes were excited with the 405 nm laser line. Both dyes were detected using a hybrid detector setting, where wavelength/bandwidth was set to 455/90 nm (Hoechst), and 530/60 nm (ELF97), respectively. The image was taken as a z-stack to capture the 3D structure of the scaffold. The image is therefore the accumulated intensity of the entire stack.

### Statistical methods

Two-way ANOVA was used to test for significant differences between groups of different time points for PCR and AlamarBlue assays. Geisser-Greenhouse correction was applied, followed by Sidak’s multiple comparisons test. Brown-Forsythe and Welch One-Way ANOVA test, corrected for multiple comparison with Dunnett T3 was employed to test for significance in groups after ARS staining. The data analysis was done using GraphPad Prism version 9.0.0 (GraphPad Software, US). Differences were considered significant when *p* *<* 0.05.

## Results and discussion

**Fig. 2:**
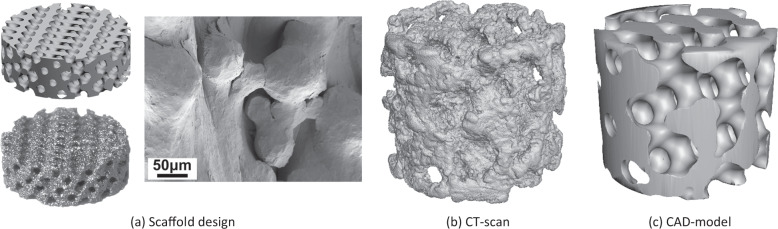
**a** Ti64-scaffolds are designed with a pore- and lattice- diameter of 800 µm. Representative images of CAD-model (top, left), EBM manufactured scaffold (bottom, left) and SEM image (right) of a large scaffold. **b** CT-scan of a manufactured small scaffold based of the CAD-model in **c**

Scaffolds with pore- and lattice- diameters of 800 µm are designed and manufactured using EBM with Ti64 alloy powder (Fig. 2a). CT-scan reveals a uniformly rough surface with partially melted particles (Fig. 2b). The scaffold has a volume and surface area of 61.6 mm^3^ and 397.4 mm^2^, respectively. In comparison, the CAD model (Fig. 2c) has a volume and surface area of 61.2 mm^3^ and 270.6 mm^2^, respectively. Overall, the manufactured part has similar volume to the CAD model. The surface area is, as expected, higher for the manufactured part. This is related to the partially melted particles from the EBM process.

**Fig. 3:**
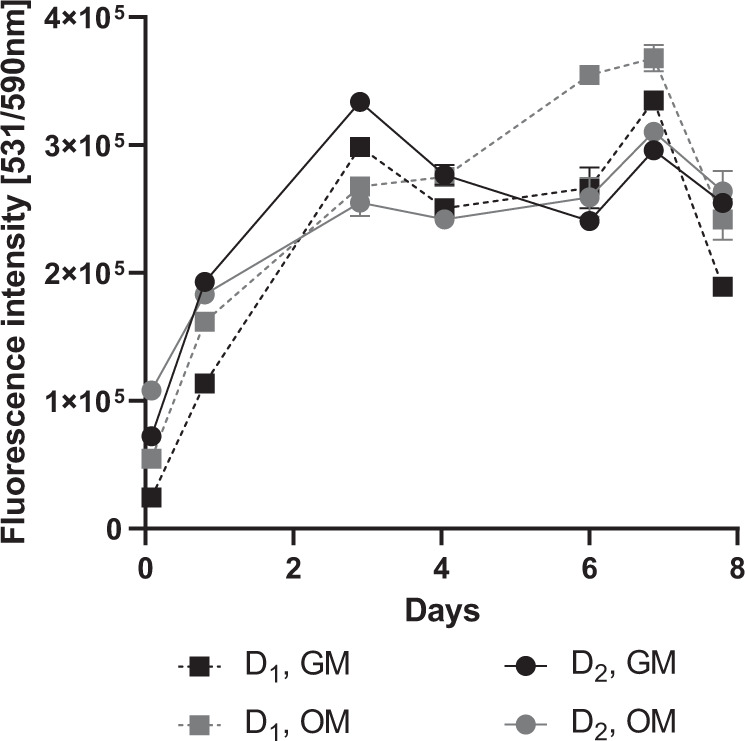
The viability of BMSCs seeded on the scaffolds cultured with growth medium (GM) or osteogenic medium (OM). The viability was measured with AlamarBlue. D_1_ Donor 1, D_2_ Donor 2

BMSCs of two donors, D_1_ and D_2_, are cultured on the scaffolds. The biocompatibility of the scaffolds is evaluated by measuring the cell viability using AlamarBlue assays for both donors in both culturing condition, GM and OM. The results indicate an increase in cell viability for both donors cultured in both media (Fig. 3). The cell viability is significantly enhanced at all time points when compared to day 0 (*p* *<* 0.002), which suggests that the cells are able to survive and proliferate when cultured on the scaffolds. From day 7 to day 8 we see a decrease in viability for all samples. This decrease is significant for D_1_ GM (*p* = 0.019) and OM (*p* = 0.023), but not for D_2_. The viability change is however not significant from day 6 to day 8. Due to this, we relate this decrease to an isolated case. Moreover, other studies have reported increasing cell viability of BMSCs throughout 21 days of culturing [25, 26]. For future research it should be considered to measure the viability for a longer period. Overall, these results correspond to studies conducted with MSCs cultured on both forged and powder metallurgical Ti64 [26], studies on differentiated MLO-A5 cell line cultured on 2D Ti64 discs [27], and MG-63 cell line cultured on laser melted Ti64 scaffolds [28]. We can conclude that increased activity on both material and 3D topology can be seen for BMSCs, corroborating with previous findings.

**Fig. 4:**
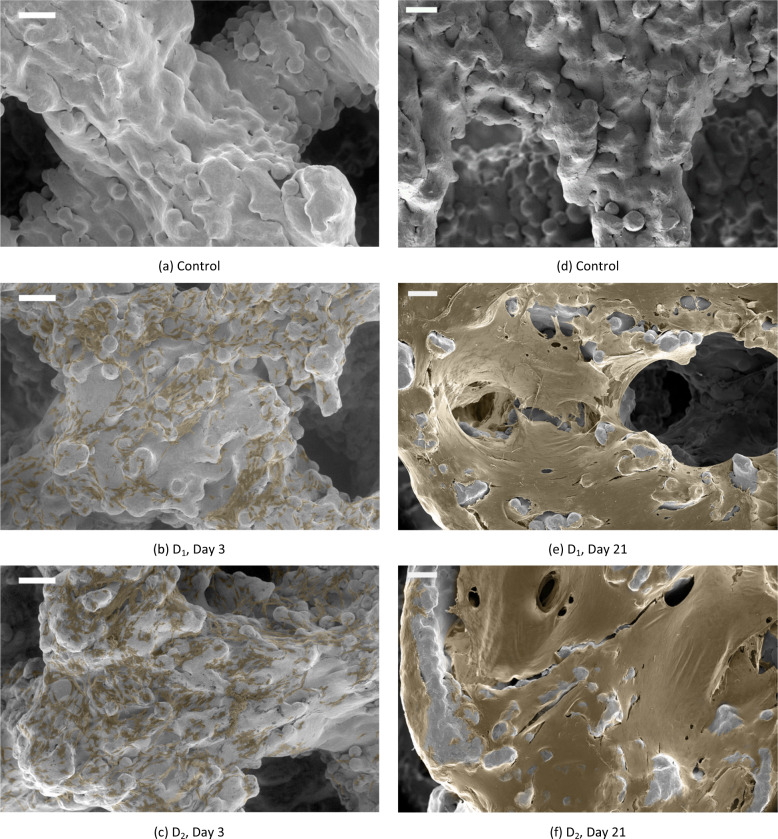
Cell adhesion and matrix development from two donors as revealed by ESEM (**a**, **b**, **c**) and SEM (**d**, **e**, **f**) imaging. Adhesion can be seen in the ESEM at day 3 (**b** and **c**), where cells are highlighted with a brown color. See cell-free control sample in (**a** and **d**) for comparison. The bone matrix development is examined using SEM at day 21 (**e** and **f**). D_1_ Donor 1, D_2_ Donor 2. Scale bar: 200 μm

The cell distribution and bone matrix development of two donors (D_1_ and D_2_) are investigated using scanning electron microscopy (SEM) and environmental SEM (ESEM). To distinguish between cells, a matrix and the substrate material, the SEM images have been colored to highlight the cells and matrix (Fig. 4). See supporting information for the original SEM images (Supplementary Fig. S1). Imaging after 3 days of culturing shows an even distribution of cells (Fig. 4b, c). The cells appear as dark particles that stand in clear contrast to the scaffold material (See control, Fig. 4a, for comparison). The distribution of the cells appears to be similar throughout the entire scaffold. Generally, the adherent cells have no preferred orientation and a highly spread morphology. After 21 days of culturing, a clear change in the number of cells and matrix development is apparent. The cells have covered the surface, and some of the pores are nearly filled with matrix (Fig. 4e, f). Only a few partially melted particles remain free of cells and matrix (See control, Fig. 3d, for comparison). These tendencies are similar to those observed by others not culturing BMSCs but osteoblast-like cells on Ti64 AM scaffolds [23, 29–32].

**Fig. 5:**
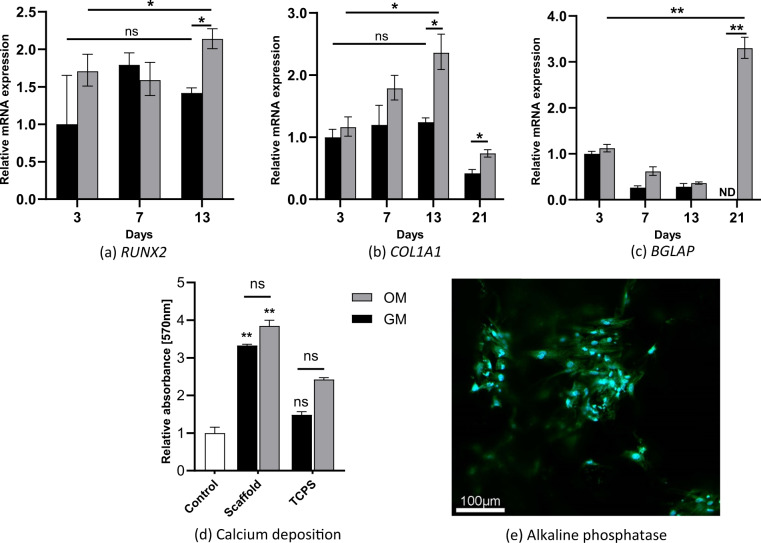
Osteogenic differentiation. mRNA expression of (**a**) *RUNX2*, (**b**) *COL1A1*, and (**c**) *BGLAP*. The results are presented relative to gene expression at day 3 for cells cultured in GM (*n* = 3), ND not detected. **d** Absorbance measurements after staining for calcium depositions at 28 days. The control sample is a cell-free control and TCPS stands for cells cultured in a tissue culture polystyrene well plate. ^∗^*p* *<* 0.05, ^∗∗^*p* *<* 0.01, ^∗∗∗^*p* *<* 0.001, ns not significant. **e** Alkaline phosphatase staining of cells cultured on the scaffolds with OM at day 7. Alkaline phosphatase is stained using ELF97 (green), and the nuclei are stained using Hoechst (blue)

Osteogenesis of the BMSCs is investigated with quantitative polymerase chain reaction (qPCR), alkaline phosphatase (ALP) staining and staining of calcium deposition, as shown in Fig. 5.

To evaluate the osteogenic differentiation, qPCR analysis is performed. Runx2 is regarded as one of the earliest markers of osteoblast differentiation. *RUNX2* expression is increased in the OM cultured cells during the culture period (day 13 vs day 3: *p* = 0.034), but not in the GM cultured cells (Fig. 5a). Additionally, after 13 days of culture, cells in OM express more *RUNX2* compared with cells cultured in GM (*p* = 0.012). This suggests that the BMSCs have differentiated towards osteoblasts.

Differentiation in the osteogenic direction is further supported by the increased mRNA expression of *COL1A1*. *COL1A1* encodes Type I collagen, an important component of the osteoid matrix [33]. *COL1A1* is expressed in all samples (Fig. 5b), and the expression increases up until 13 days of culture for cells cultured in OM (day 13 vs day 3: *p* = 0.023). Expression of *COL1A1* in cells cultured in GM from day 3 to day 13 is not increased. These results concur with other studies, where *COL1A1* expression remains constant for BMSCs cultured with regular GM, and increase expression when cultured with OM over time [25, 34].

This indicates that the osteoblasts can produce osteoid, the unmineralized portion of the bone matrix.

Osteocalcin (encoded by *BGLAP*) is involved in the process of mineralization [35]. *BGLAP* is expressed in all samples, except for cells cultured in GM at day 21 (Fig. 5c). The expression is significantly increased for cells cultured in OM at day 21 compared to day 3 (*p* = 0.009). This suggests that the bone matrix is in the process of mineralization.

The extent of calcium deposition gives further insight into the progression of mineralization of the bone matrix. The quantification of calcium deposits after 28 days of culturing is shown in Fig. 5d. Calcium deposition is detected in both OM and GM cultured cells (*p* *<* 0.001). Images from light microscopy after staining are shown in supplementary material, S3 Calcium deposition. The calcium is evenly distributed throughout the scaffolds in both culturing conditions, which corresponds to the absorbance measurements. These results suggest that the bone is mineralized.

We see a significant amount of calcium deposition on cells cultured on scaffolds in both GM and OM. It was shown by researchers Choi et al. that culturing in ascorbic acid (AA) results in higher proliferation rate and higher calcium content [36]. The high amount of AA in the basal medium can thus explain the presence of calcium in the GM sample. However, for cells cultured in GM on TCPS, we do not detect significant amount of calcium. Based on this, we hypothesize that the surface and material of EBM Ti64 are able to induce matrix mineralization in terms of calcium deposition.

ALP activity in osteoblasts indicates the onset of mineralization of the bone matrix. The ALP activity, shown with confocal imaging (Fig. 5e), shows that the mineralization had started by day 7 for cells cultured in OM. ALP was not detected for cells cultured in GM.

To see if the osteoblasts terminally differentiated into osteocytes, the mRNA expression of *SOST* is analyzed. *SOST* is expressed solely by osteocytes and encodes for the protein sclerostin. No expression of sclerostin is detected during the culturing period. Possible explanations are: (1) At a low number of cells, one of the fates of osteoblasts is to undergo apoptosis after bone formation [37], which, in turn, reduces the amount of cells. Alternatively, osteoblasts are known to get buried in the matrix, causing them to terminally differentiate to osteocytes. This introduces the next interpretation: (2) The inability to lyse the osteocytes; since the matrix at day 21 appeared to be thick, an unsuccessful lysis of osteocytes could be a possibility. The low amount of mRNA at day 21 also supports that the cells are not properly lysed (Supplementary Fig. S2). (3) The osteocytes did not reach their mature stage; it has been shown that sclerostin is only produced by mature osteocytes [38], necessitating a prolonged culturing period. (4) Finally, a combination of the above may also explain our observation.

Taken together, the gene expression data supports that the cells cultured in OM on the scaffolds are able to differentiate into osteoblasts. Imaging shows large amounts of bone matrix and staining of calcium suggests that the matrix is mineralized. The terminal differentiation to osteocytes could however not be revealed, and a prolonged culturing period might be necessary.

## Conclusion

From the obtained results, the following conclusions can be drawn:

1. Untreated electron beam melted Ti64 scaffolds provide an environment that is biocompatible facilitating differentiation of BMSCs into osteoblasts.
2. qPCR analysis suggests that the BMSCs differentiated into osteoblasts.
3. Imaging reveals an even distribution of cells after 3 days of culturing and high matrix development after 21 days of culturing.
4. Staining of alkaline phosphatase at day 7 and calcium deposits at day 28 suggest that the bone matrix is mineralized.

## Supplementary information

Supplementary Information

Supplementary figure
