# Supplementary material for: Revealing the influence of electron beam melted Ti-6Al-4V scaffolds on osteogenesis of human bone marrow-derived mesenchymal stromal cells
Source: J Mater Sci Mater Med. 2021 Aug 18;32(9):97. doi: 10.1007/s10856-021-06572-0 (PMC8373740; doi:10.1007/s10856-021-06572-0)
Supplement: Supplementary file 1 — Supplementary Information [file 10856_2021_6572_MOESM1_ESM.docx]

Supporting Information

**Revealing the influence of electron beam melted Ti-6Al-4V scaffolds on osteogenesis of human bone marrow-derived mesenchymal stromal cells**

*Kristin S. Ødegaard^1^, Lingzi Ouyang^2^, Qianli Ma^2^, Glenn Buene^2^, Di Wan^1^, Christer W. Elverum^1^, Jan Torgersen^1^*, Therese Standal^2^, and Marita Westhrin^2^*

^1^ Department of Mechanical and Industrial Engineering, Norwegian University of Science and Technology, Norway

^2^ Department of Clinical and Molecular Medicine, Norwegian University of Science and Technology, Norway

* E-mail: [jan.torgersen@ntnu.no](mailto:jan.torgersen@ntnu.no)

**Contents**

[S1 Imaging 2](#_Toc75440185)

[S2 mRNA concentrations 3](#_Toc75440186)

[S3 Calcium deposition 4](#_Toc75440187)

**Figures**

[Figure S 1: Cell adhesion and matrix development. 2](#_Toc75440193)

[Figure S 2: mRNA concentrations. 3](#_Toc75440194)

[Figure S 2: Results from calcium deposition staining. 4](#_Toc75440195)

# Imaging

(a) Control

(d) Control

(b) D_1_, Day 3


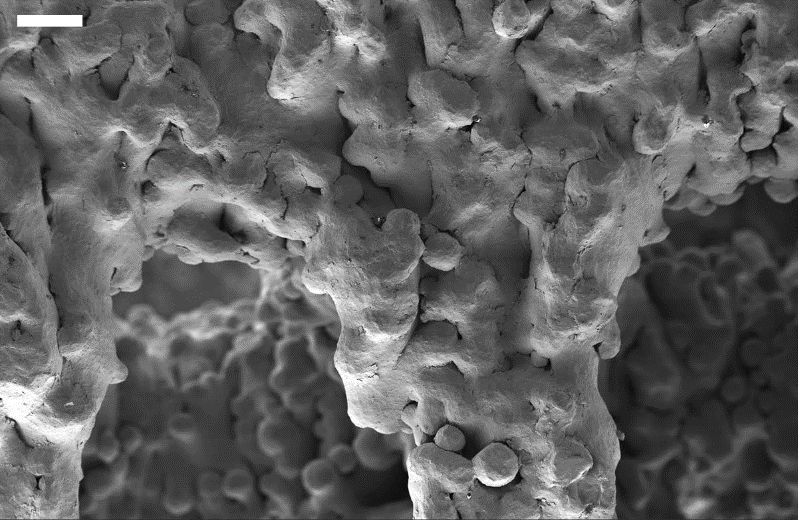

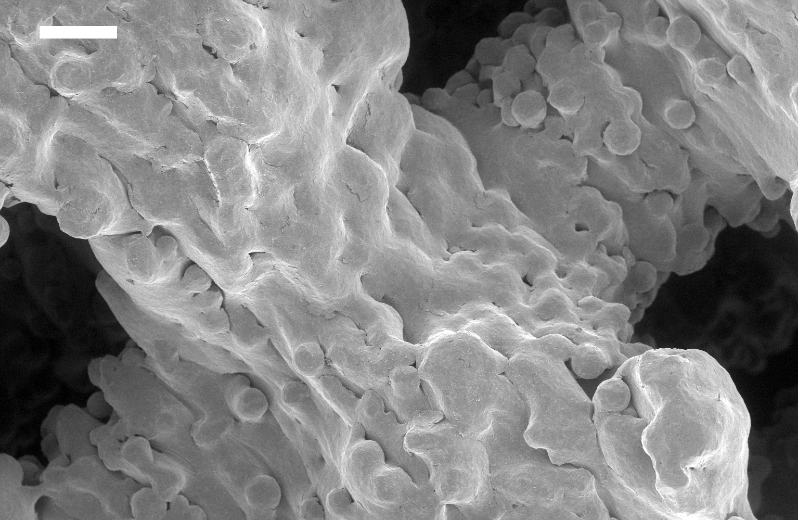


(e) D_1_, Day 21

(f) D_2_, Day 21

(c) D_2_, Day 3


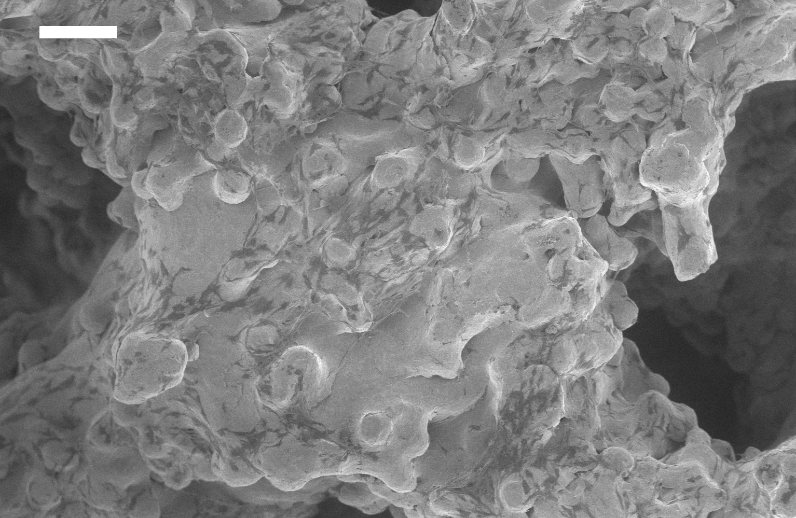

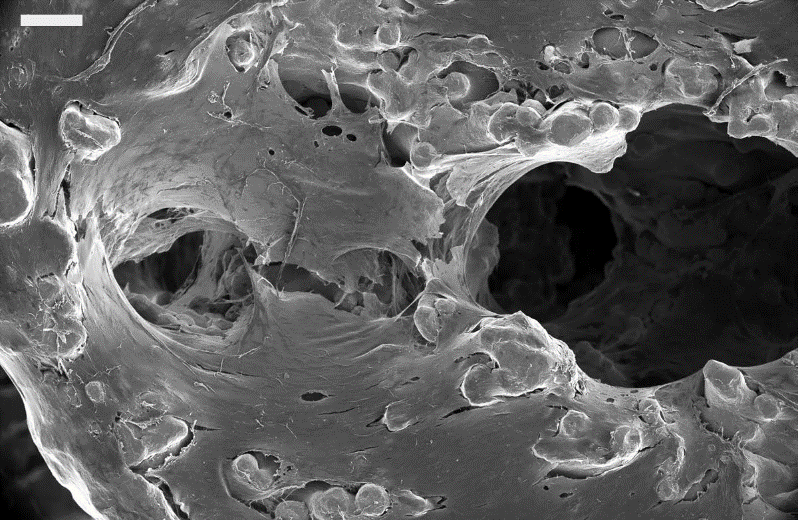

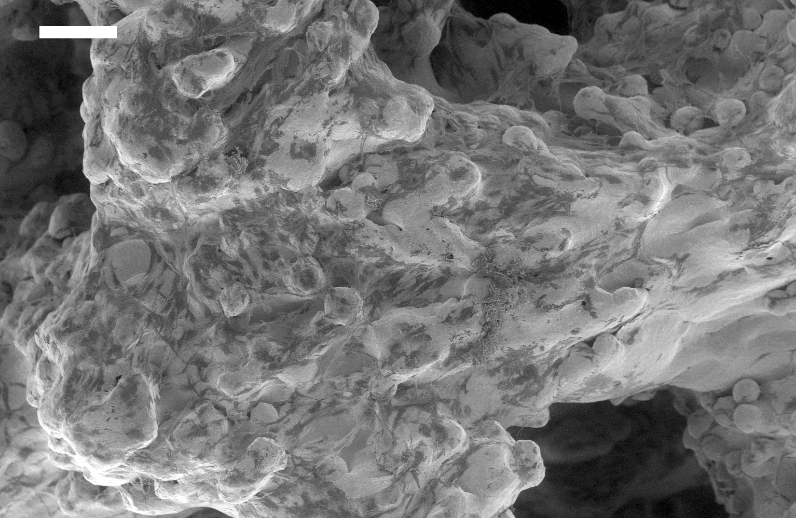

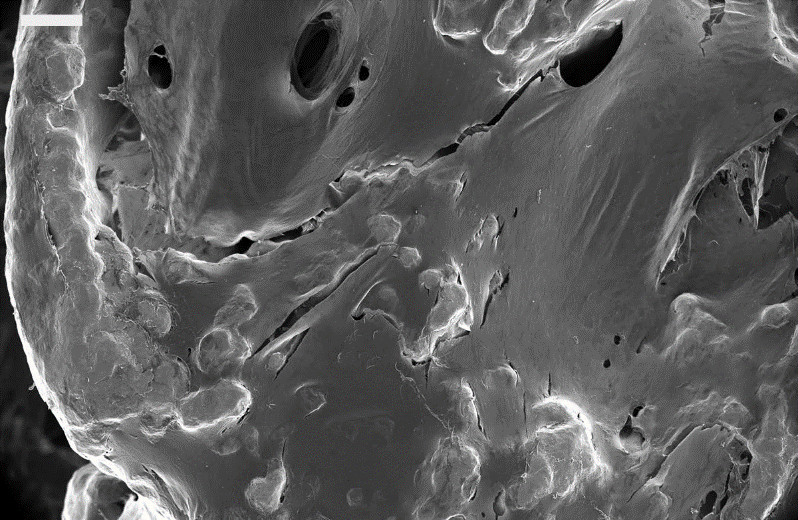


Figure S 1: Cell adhesion and matrix development from two donors as revealed by ESEM (a,b,c) and SEM (d,e,f) imaging. Adhesion can be seen in the ESEM at day 3 (b and c), where cells appear as dark particles that stand in clear contrast to the scaffold material, see cell-free control sample in (a and d). The bone matrix development is examined using SEM at day 21 (e and f). D_1_: Donor 1, D_2_: Donor 2. Scale bar: 200 μm

# mRNA concentrations


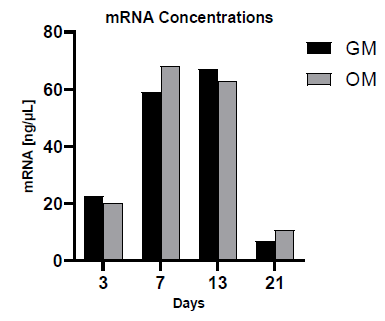


Figure S 2: mRNA concentrations. Cells at designated days were lysed directly on the scaffolds. mRNA was isolated using an RNA isolation kit, and concentrations were measured using a spectrophotometer.

# Calcium deposition

BMSCs from one donor were cultured on small scaffolds for 28 days in two culturing conditions: OM and GM. The scaffolds were then stained for calcium deposits, as described in S1.6 Calcium deposition. A cell-free scaffold was used as control.

Figure S3 shows the light microscopy images taken after the staining. The images were taken with an inverted light microscope. Here we see a deeper stain on the scaffolds cultured with cells (Figure S3b and c) compared to the control scaffold (Figure S3a). This corroborates the results from the absorbance reading. It is however difficult to distinguish between the two culturing conditions. This can be related to the scaffold structure and the amount of matrix and ARS intesity on the scaffolds (Figures S3b and S3c), which inhibited light to pass through.


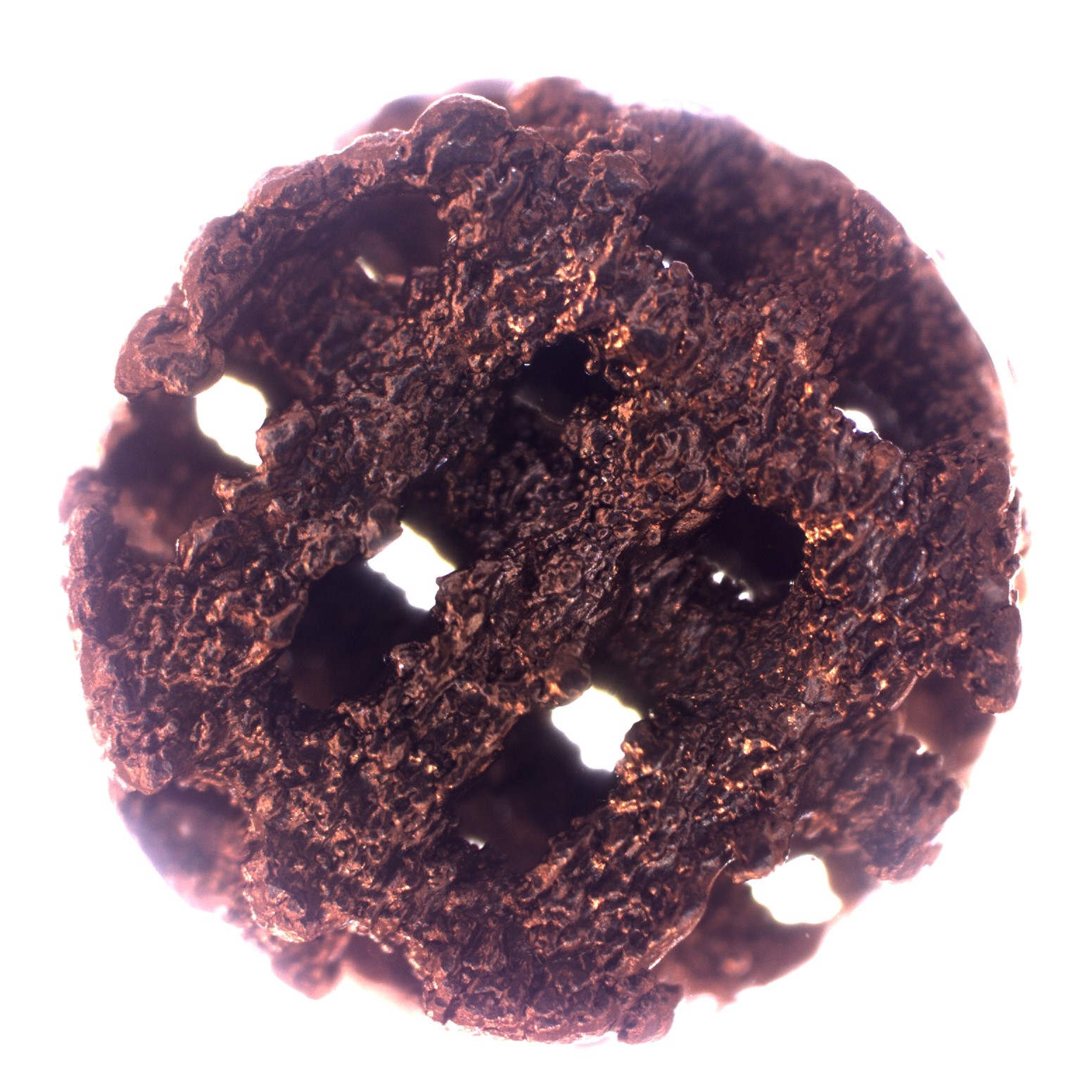

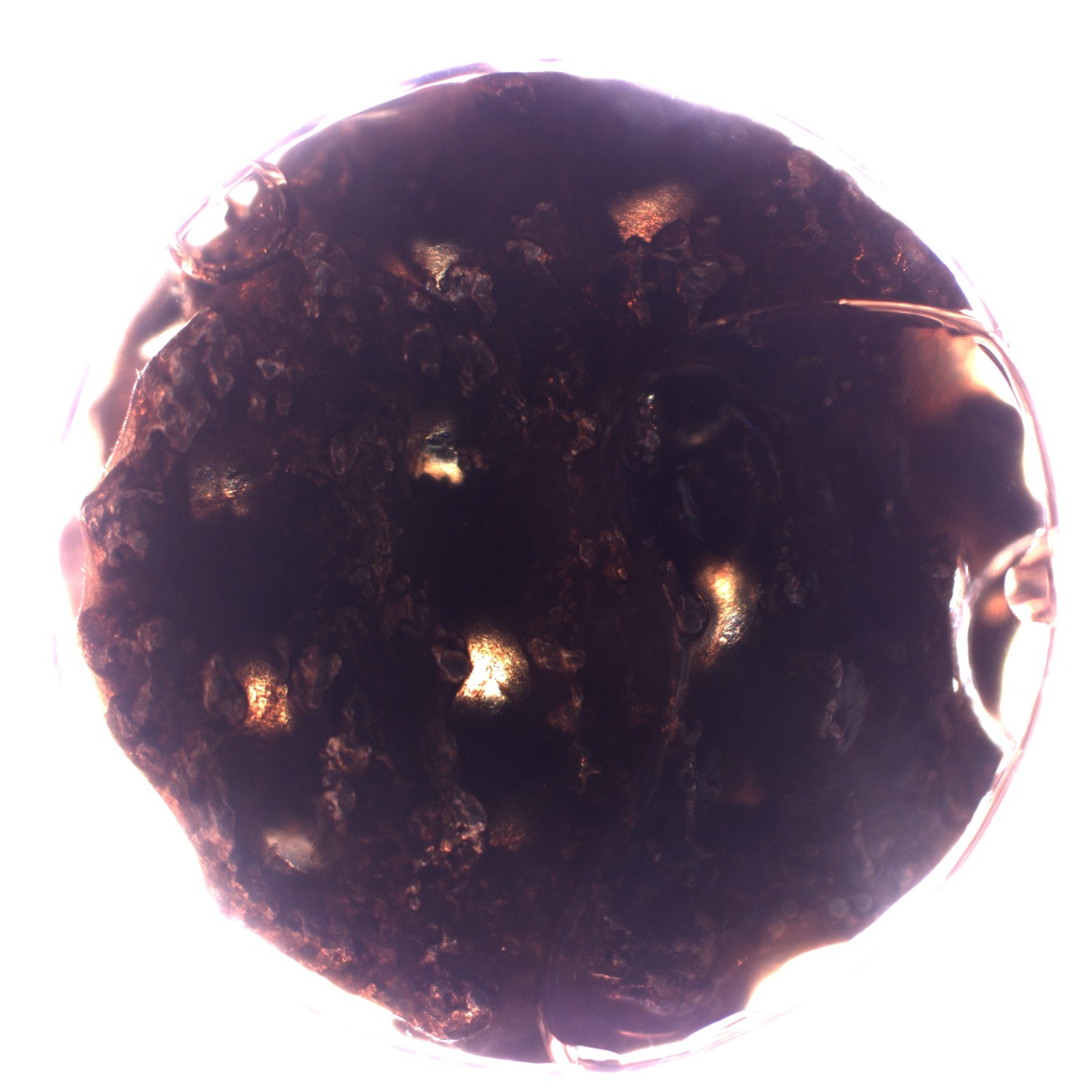

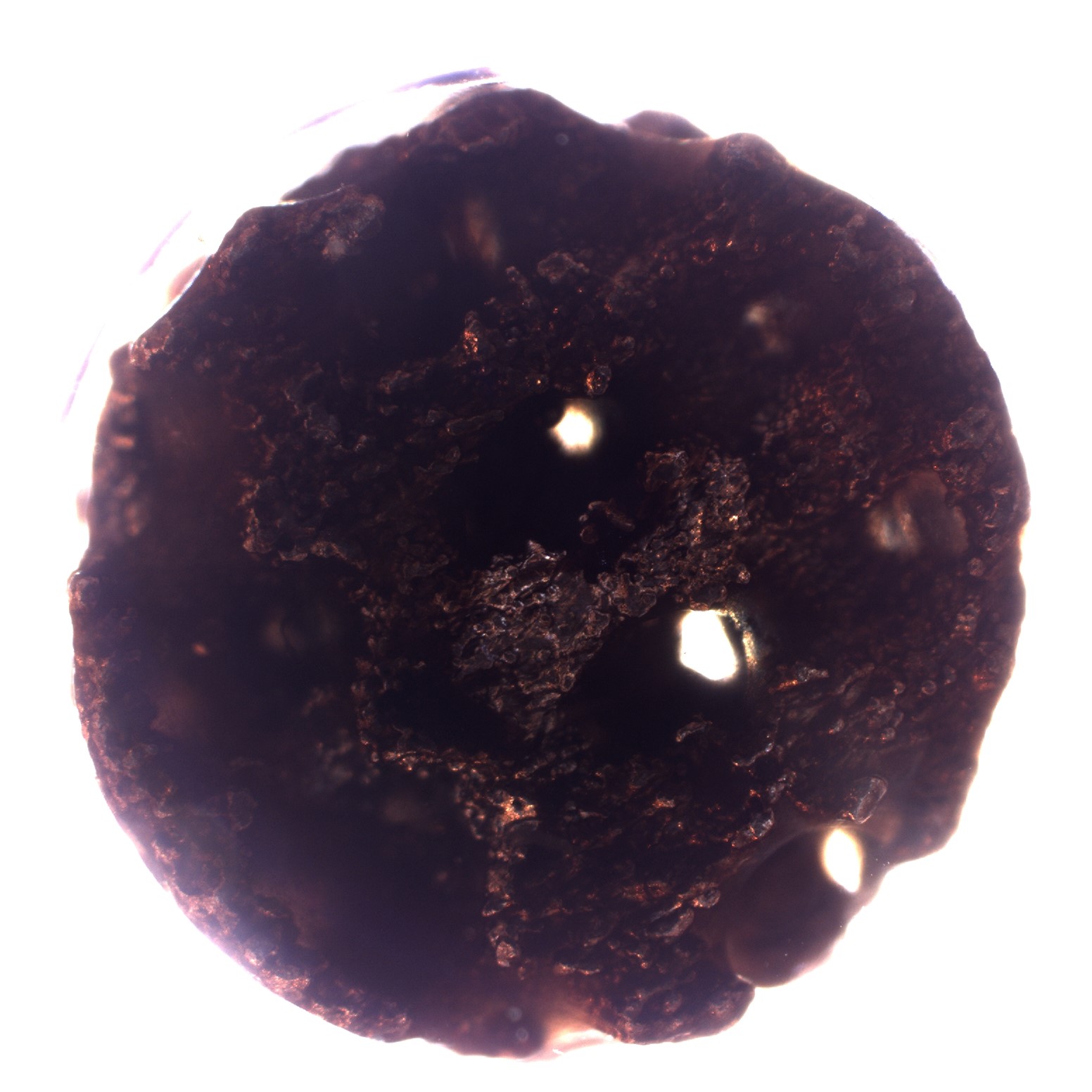


(a) Control (b) OM (c) GM

Figure S 3: Results from calcium deposition staining.
